# The small noncoding RNAs (sncRNAs) of murine gammaherpesvirus 68 (MHV-68) are involved in regulating the latent-to-lytic switch *in vivo*

**DOI:** 10.1038/srep32128

**Published:** 2016-08-26

**Authors:** Beatrix Steer, Martin Strehle, Christine Sattler, Dagmar Bund, Britta Flach, Tobias Stoeger, Jürgen G. Haas, Heiko Adler

**Affiliations:** 1 Comprehensive Pneumology Center, Research Unit Lung Repair and Regeneration, Helmholtz Zentrum München - German Research Center for Environmental Health (GmbH), Member of the German Center of Lung Research (DZL), Marchioninistrasse 25, D-81377 Munich, Germany; 2 Institute of Lung Biology and Disease, Comprehensive Pneumology Center, Helmholtz Zentrum München - German Research Center for Environmental Health (GmbH), Member of the German Center of Lung Research (DZL), Ingolstädter Landstrasse 1, D-85764 Neuherberg, Germany; 3 Division of Infection and Pathway Medicine, University of Edinburgh, 49 Little France Crescent, Edinburgh EH16 4SB, UK

## Abstract

The human gammaherpesviruses Epstein-Barr virus (EBV) and Kaposi’s sarcoma-associated herpesvirus (KSHV), which are associated with a variety of diseases including tumors, produce various small noncoding RNAs (sncRNAs) such as microRNAs (miRNAs). Like all herpesviruses, they show two stages in their life cycle: lytic replication and latency. During latency, hardly any viral proteins are expressed to avoid recognition by the immune system. Thus, sncRNAs might be exploited since they are less likely to be recognized. Specifically, it has been proposed that sncRNAs might contribute to the maintenance of latency. This has already been shown i*n vitro*, but the respective evidence *in vivo* is very limited. A natural model system to explore this question *in vivo* is infection of mice with murine gammaherpesvirus 68 (MHV-68). We used this model to analyze a MHV-68 mutant lacking the expression of all miRNAs. In the absence of the miRNAs, we observed a higher viral genomic load during late latency in the spleens of mice. We propose that this is due to a disturbed regulation of the latent-to-lytic switch, altering the balance between latent and lytic infection. Hence, we provide for the first time evidence that gammaherpesvirus sncRNAs contribute to the maintenance of latency *in vivo*.

Long and small noncoding RNAs (ncRNAs) including microRNAs (miRNAs) produced by eukaryotic cells contribute to a variety of cellular processes like gene expression and genome maintenance^1^. Similar to their host cells, many viruses also produce ncRNAs. Among the viruses, herpesviruses produce most ncRNAs which might be related to their large genomes and their particular life cycle^2^, consisting of a lytic and a latent phase. In the lytic phase, all viral gene products are expressed and infectious progeny is produced while in the latent phase, the double-stranded DNA genome is maintained in the host cell but only a few viral gene products are expressed^2^. Therefore, during latency, the use of ncRNAs like miRNAs instead of viral proteins for the regulation of viral persistence might be of advantage for the virus as ncRNAs are less likely to be recognized by the host immune system^2^.

Great efforts have been made to unravel the targets and the functions of viral ncRNAs, particularly of virus-encoded miRNAs. The latter are approximately 22 nucleotide-long ncRNAs, which are generated from stem-loop precursors. Mature miRNAs directly interact with a member of the Argonaute protein family to form the RNA-induced-silencing-complex (RISC), which silences gene expression post-transcriptionally by binding to the 3′ untranslated regions of target mRNAs (reviewed, for example, in^3^,^4^,^5^). During the past years, so-called ribonomics approaches for miRNA target analysis, for example RIP-Chip (RNA immunoprecipitation coupled with microarray analyses), CLIP (crosslinking and immunoprecipitation), HITS-CLIP (high-throughput sequencing of crosslinked RNA fragments) and PAR-CLIP (photoactivatable-ribonucleotide-enhanced crosslinking)^6^ have been applied and led to the identification of numerous herpesviral miRNA targets^7^,^8^,^9^,^10^. Besides the identification of potential targets of a herpesviral miRNA, the demonstration of its biological function, preferentially in the context of viral infection, is needed to finally define its role in infection and pathogenesis^11^. Using various model systems combined with reverse genetics approaches, important roles for herpesviral ncRNAs in the viral life cycle and in pathogenesis have been described, including the regulation of viral persistence, host cell proliferation, long-term survival of host cells and immune evasion (reviewed, for example, in^11^,^12^,^13^,^14^,^15^,^16^,^17^,^18^,^19^,^20^,^21^,^22^). However, the biological functions have so far largely been explored in *in vitro* systems whereas the respective knowledge during infection *in vivo* is still very limited. To understand the biological relevance, *in vivo* systems are essential^23^,^24^. In this regard, progress has recently been made for miRNAs encoded by different members of all three herpesvirus subfamilies. The human alphaherpesviruses herpes simplex virus (HSV)-1 and HSV-2 are able to also infect various animals and can thus be studied *in vivo*. Mutation of the miRNA hsv1-miR-H2 of HSV-1 led to increased encephalitis in mice and to increased reactivation from latency *ex vivo*^25^. However, a combined knockout of miRNAs hsv1-miR-H2, -H4, -H5 and -H6 did not affect replication and levels of latent virus in infected mice^26^. The miRNA hsv2-miR-H6 of HSV-2 was shown to contribute to pathogenesis in guinea pigs: Disruption of hsv2-miR-H6 resulted in attenuation of neurological symptoms while it did not affect replication or levels of latent virus^27^. Pseudorabies virus (PRV) is an alphaherpesvirus of pigs, thus, experimental infection of pigs represents a robust natural model to investigate the *in vivo* function of miRNAs. Deletion of 9 of the 11 PRV miRNAs did neither affect replication nor latency establishment in infected pigs^28^. Marek’s disease virus-1 (MDV-1) is an avian alphaherpesvirus, hence, as with PRV infection of pigs, infection of chickens represents a very good natural model to investigate the *in vivo* function of miRNAs. Using this model, Zhao *et al*. demonstrated a critical role for mdv1-miR-M4, a functional ortholog of the cellular oncomir miR-155, in MDV-1 induced lymphomagenesis^29^, and Teng *et al*. demonstrated that additional MDV-1 encoded miRNAs also contribute to tumorigenesis^30^. For the human betaherpesvirus human cytomegalovirus (HCMV) which encodes 17 mature miRNAs, infection of mice with murine cytomegalovirus (MCMV) offers a model to study the *in vivo* function of viral miRNAs in a natural host^23^. Mutation of two (mcmv-miR-M23-2 and mcmv-miR-m21-1) out of the 29 mature miRNAs encoded by MCMV resulted in reduced salivary gland replication in C57BL/6 mice due to compromised immune evasion^31^. The human gammaherpesviruses Epstein-Barr virus (EBV) and Kaposi’s sarcoma-associated herpesvirus (KSHV) encode for 44 and 25 mature miRNAs, respectively^23^. *In vivo* investigation of their role is difficult due to the strict species specificity of both viruses. Nevertheless, by using humanized mice, insights into *in vivo* functions of EBV and KSHV miRNAs could be obtained. For example, an EBV mutant with a deletion of the three BHRF-1 miRNAs displayed a delay in systemic viral DNA accumulation in humanized mice but did not affect virus-induced oncogenesis^32^. Xenotransplantation of EBV-negative cells ectopically expressing EBV miR-BART7-3p increased the development of metastases^33^, and ectopic expression of all BART miRNAs potentiated tumor growth in a mouse xenograft model^34^. Similarly, transplantation of human hematopoietic progenitors stably expressing KSHV miR-K12-11, an ortholog of the cellular miR-155^35^,^36^, into immunodeficient mice resulted in a strong B-cell expansion^37^,^38^.

A natural model system to investigate gammaherpesvirus-host interaction including the *in vivo* function of gammaherpesvirus miRNAs is the infection of mice with murine gammaherpesvirus 68 (MHV-68), a natural pathogen of wild rodents^39^. MHV-68 encodes for 14 pre-miRNA stem-loops giving rise to 28 mature miRNAs^40^,^41^,^42^. The stem-loop sequences are incorporated into 8 viral tRNA (vtRNA) sequences which are under the regulation of RNA-Polymerase III. It has been shown that the generation of the MHV-68 miRNAs is dependent on the A/B boxes in these vtRNA sequences and on the presence of tRNase Z and Dicer, but not on Drosha^43^,^44^. Uncovering the function of these vtRNA-miRNA-encoding sequences has only just started. Feldman *et al*.^45^ and Diebel *et al*.^46^ demonstrated that after intranasal inoculation of C57BL/6 wildtype mice, mutants lacking the expression of all miRNAs undergo normal acute lytic replication in lungs but show a subtle latency phenotype, reflected by a slightly reduced latency load at early latency (around two weeks after infection). Notably, the mutants displayed a striking attenuation of lethal pneumonia in Balb/c IFN-γ^−/−^ mice.

Here, we analyze an independently generated MHV-68 mutant lacking the expression of all miRNAs in several animal models including C57BL/6 wildtype mice and IFN-γ-R^−/−^ mice. First, we test whether our mutant would display a phenotype consistent with the previous findings of Feldman *et al*.^45^ and Diebel *et al*.^46^. This was crucial since the mutants have been generated in different ways. Second and most importantly, we extend the previous studies to the phase of late latency (around six weeks after infection). We show that during late latency, the absence of the sncRNAs results in a higher viral genomic load in the spleen. Hence, we provide for the first time evidence that gammaherpesvirus sncRNAs contribute to the maintenance of latency *in vivo*.

## Results

### Generation of MHV68 vtRNA/miRNA knockout viruses

**Figure 1:**
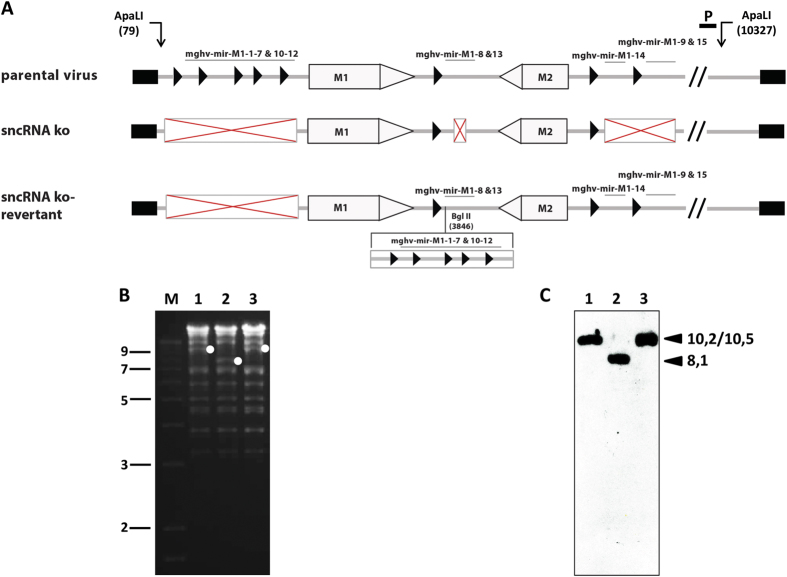
Construction and characterization of the sncRNA ko and sncRNA ko-revertant. (**A**) Scheme of the recombinant viruses generated. The boxed red “X” schematically represent the deletions and the black triangles (▶) the tRNAs. (**B**) Structural analysis of BAC plasmids by ethidium bromide-stained agarose gel analysis of *ApaL*I-digested DNA. The lanes show parental BAC (lane 1), sncRNA ko BAC (lane 2) and sncRNA ko-revertant BAC (lane 3). Marker (M) sizes (in kilobase pairs) are indicated on the left. (**C**) Southern blot analysis of the gel shown in panel B using a DIG-labeled probe corresponding to nucleotides 9340 to 10104 (indicated as “P” in panel A). The white dots in panel B and the arrowheads in panel C indicate the expected fragments of 10,2 kb, 8,1 kb and 10,5 kb for parental BAC, sncRNA ko BAC and sncRNA ko-revertant BAC, respectively.

**Figure 2:**
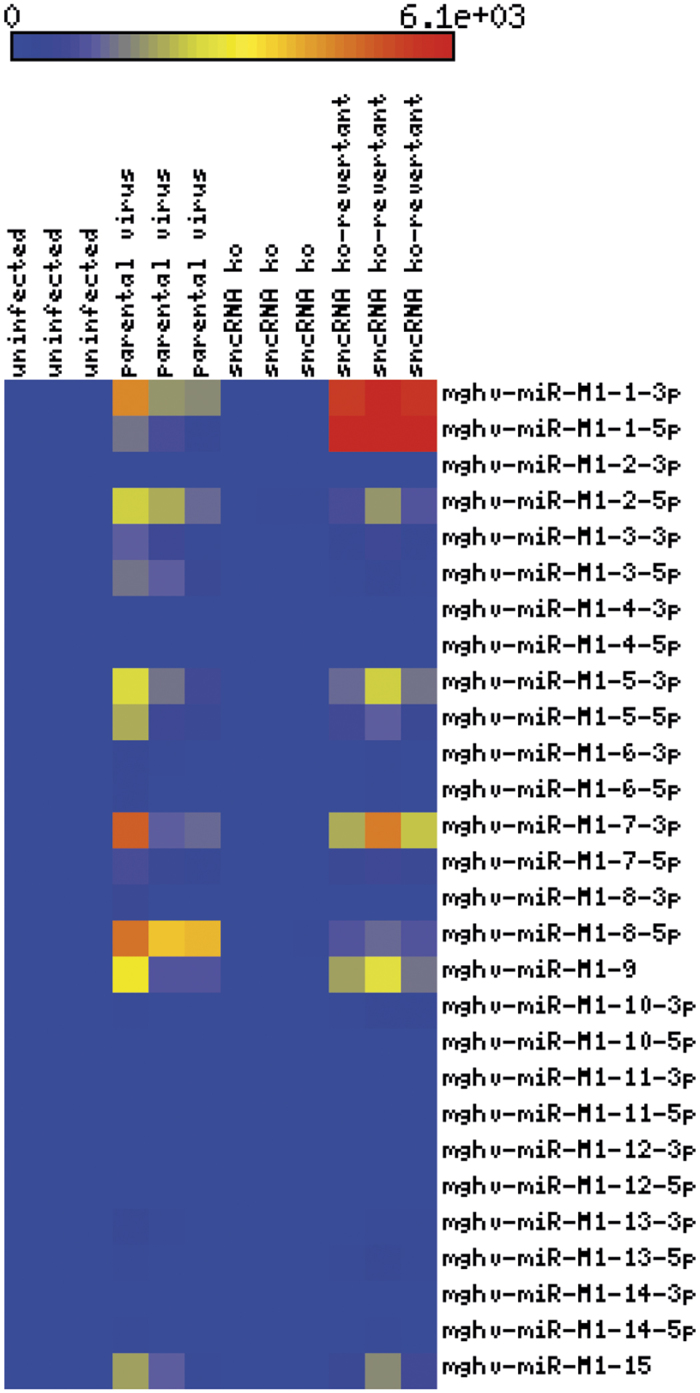
miRNA expression analysis. NIH3T3 cells were cultured in 6-well plates and infected with the indicated viruses at an MOI of 1, or were left uninfected. For each condition, three biological replicates were done. Total RNA was isolated 48 hr p.i., and the analysis of the expression of the MHV-68 miRNAs was performed by LC Sciences (Houston, Texas, USA) using custom miRNA microarrays. The heatmap was generated using the matrix2png web interface^64^.

As explained above, the vtRNAs and miRNAs of MHV-68 are very tightly linked. As described in the following, we do not differentiate between the vtRNAs and miRNAs, and therefore refer to the vtRNA-miRNA-encoding sequences as “sncRNAs” in our manuscript. To investigate the function of the sncRNAs of MHV-68, we constructed a mutant lacking all known sncRNAs (designated as sncRNA ko) using MHV-68 cloned as bacterial artificial chromosome (BAC)^47^. To exclude the possibility that the observed results were due to rearrangements outside the mutated regions, a revertant of the sncRNAs ko mutant (designated as sncRNA ko-revertant) was also generated. The recombinant MHV-68 used in this study are schematically depicted in Fig. 1A. All BAC plasmids were characterized by restriction enzyme analysis (Fig. 1B), Southern blot analysis (Fig. 1C) and by sequencing across the mutated regions which all yielded the expected results. To exclude that the genome manipulations affected neighbouring genes, transcription of open reading frames M1, M2 and M3 was determined by quantitative RT-PCR 48 hours after infection of NIH3T3 cells with parental virus, sncRNA ko and sncRNA ko-revertant, respectively. No significant differences were observed (Supplementary Figure S1). Finally, the expression of all known MHV-68 encoded miRNAs was assessed. For this purpose, total RNA was isolated 48 hr after infection of NIH3T3 cells, and the analysis of the expression of the MHV-68 miRNAs was performed by LC Sciences (Houston, Texas, USA) using custom miRNA microarrays. Similar as in case of cellular miRNAs, some precursor miRNAs (miR-M1-2) only led to the expression of one mature miRNA isoform in parental MHV-68 infection. No mature miR-M1-4, miR-M1-10, miR-M1-11 and miR-M1-12 miRNAs were detected in these cells after lytic infection. As expected, no MHV-68 miRNA expression at all was detected after infection with the sncRNA ko while parental virus and sncRNA ko-revertant displayed a very similar pattern of miRNA expression (Fig. 2). We conclude from these data that the sncRNA ko and sncRNA ko-revertant represent useful tools to investigate the function of the sncRNAs of MHV-68 both *in vitro* and *in vivo*.

### Lytic replication *in vitro*

**Figure 3:**
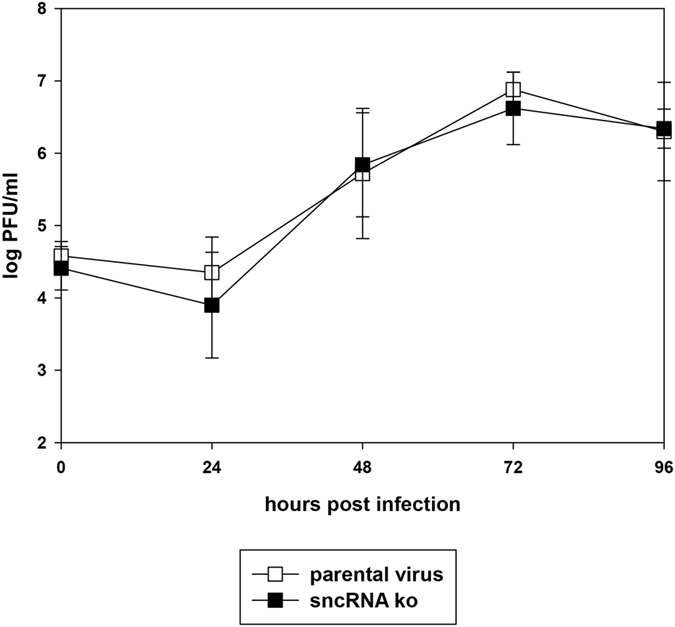
Lytic virus replication *in vitro*. NIH3T3 cells were infected with the indicated viruses at an MOI of 0.1. Cells and cell culture supernatants were harvested at different time points after infection, and virus titers were determined by plaque assay on BHK-21 cells. Data shown are the means ± SD from three independent experiments.

To analyze whether deletion of the MHV-68 sncRNAs has any effect on lytic replication *in vitro*, NIH3T3 cells were infected with sncRNA ko and parental virus, respectively, and virus titers at different time points after infection were determined. No significant differences were observed (Fig. 3), indicating that the sncRNAs are not needed for lytic replication in NIH3T3 cells *in vitro*.

### Lytic replication *in vivo* in C57BL/6 wildtype mice

**Figure 4:**
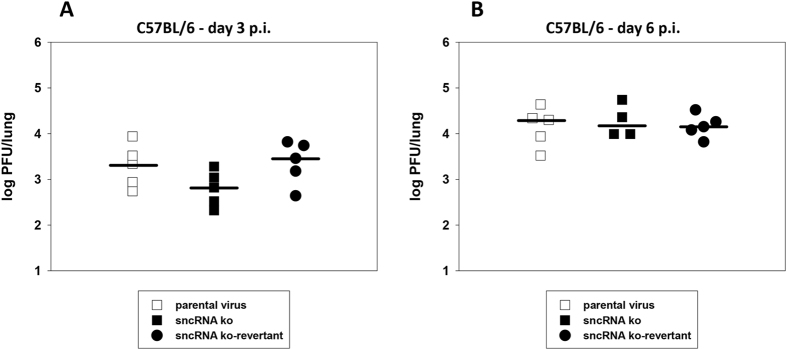
Lytic replication in the lungs of C57BL/6 mice. Mice were inoculated i.n. with 5 × 10^4^ PFU of the indicated viruses. Lungs of mice were harvested at day 3 (**A**) and at day 6 (**B**) post infection (p.i.). Virus titers were determined from organ homogenates by plaque assay. Each symbol represents an individual mouse, and the bars represent the median. The data are compiled from two independent experiments.

Intranasal (i.n.) inoculation of mice results in an acute phase of lytic virus replication in the lung which mainly involves alveolar epithelial cells^39^. Thus, to analyze whether deletion of the MHV-68 sncRNAs has an effect on lytic replication *in vivo*, C57BL/6 mice were inoculated i.n. with 5 × 10^4^ PFU of parental virus, sncRNA ko and sncRNA ko-revertant, respectively. Lungs of mice were harvested at day 3 (Fig. 4A) and at day 6 (Fig. 4B) after infection, and virus titers were determined from organ homogenates by plaque assay. No significant differences were observed, indicating that the sncRNAs are dispensable for lytic replication in the lungs of C57BL/6 mice.

### Early latency in C57BL/6 wildtype mice

**Figure 5:**
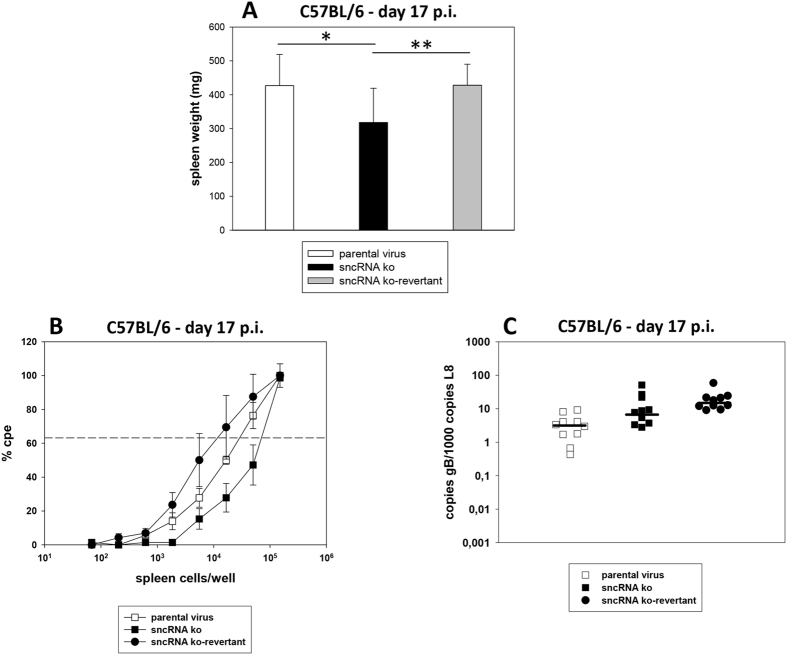
Early latency in the spleen of C57BL/6 mice. Mice were inoculated i.n. with 5 × 10^4^ PFU of the indicated viruses. At day 17 after infection, spleens were harvested, and the spleen weights were taken. Single splenocyte suspensions were prepared and analyzed in the *ex vivo* reactivation assay or used for DNA isolation for real-time PCR analysis. (**A**) Spleen weights; (**B**) *ex vivo* reactivation of splenocytes; (**C**) viral genomic load in the spleen. Data shown in panel A are the means + standard deviations of 10 mice per group, compiled from three independent experiments. The asterisks indicate a statistically significant difference: *P = 0.021; **P = 0.009 (Student’s t-test). Data shown in panel B are the means ± standard errors of the means pooled from three independent experiments. In each experiment, splenocytes from two to five mice per group were pooled. The dashed line in panel B indicates the point of 63.2% Poisson distribution, determined by nonlinear regression, which was used to calculate the frequency of cells reactivating lytic replication. In panel C, each symbol represents an individual mouse, and the bars represent the median. The data are compiled from three independent experiments.

After i.n. inoculation, the spleen is a major site of latently infected cells, and the establishment of latency is accompanied by a transient splenomegaly^39^,^48^. Thus, spleen weight, *ex vivo* reactivation of latently infected splenocytes and the viral genomic load in the spleen were determined 17 days after infection (early latency). At this stage, the majority of cells in the spleen harbouring MHV-68 are B cells^39^. Spleens were harvested and the spleen weights were taken. Single splenocyte suspensions were prepared and analyzed in the *ex vivo* reactivation assay or used for DNA isolation for real-time PCR analysis. After infection with the sncRNA ko, moderately but significantly reduced spleen weights were observed, when compared to infection with parental virus or sncRNA ko-revertant (Fig. 5A). Likewise, *ex vivo* reactivation was slightly reduced after infection with the sncRNA ko, with frequencies of reactivation being 1 in 29653, 1 in 70091 and 1 in 12144 for parental virus, sncRNA ko and sncRNA ko-revertant, respectively (P = 0.086 for parental virus versus sncRNA ko, and P = 0.030 for sncRNA ko versus sncRNA ko-revertant; Student’s t-test) (Fig. 5B). In contrast, the viral genomic load in the spleen was not reduced after infection with the sncRNA ko (P = 0.026 for parental virus versus sncRNA ko, and P = 0.089 for sncRNA ko versus sncRNA ko-revertant; Mann-Whitney Rank Sum Test) (Fig. 5C). We conclude from these data that the sncRNAs of MHV-68 somewhat contribute to splenomegaly and *ex vivo* reactivation but not to the overall genomic load in the spleen at early latency.

### Late latency in C57BL/6 wildtype mice

**Figure 6:**
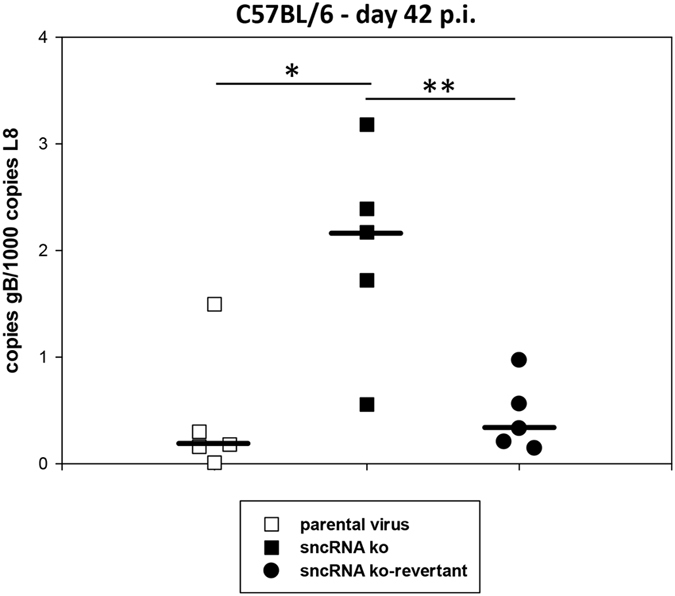
Late latency in the spleen of C57BL/6 mice. Mice were inoculated i.n. with 5 × 10^4^ PFU of the indicated viruses. At day 42 after infection, spleens were harvested and used for DNA isolation for real-time PCR analysis to determine the viral genomic load. Each symbol represents an individual mouse, and the bars represent the median. The data are compiled from two independent experiments. The asterisks indicate a statistically significant difference: *P = 0.015; **P = 0.009 (Student’s t-test).

Next, we analyzed the viral genomic load at day 42 post infection (late latency). Interestingly, mice infected with the sncRNA ko displayed a significantly higher viral genomic load when compared to infection with parental virus or sncRNA ko-revertant (P = 0.015 for parental virus versus sncRNA ko, and P = 0.009 for sncRNA ko versus sncRNA ko-revertant; Student’s t-test) (Fig. 6). The increase in viral genomic load was, however, not accompanied by an increased spleen weight or by increased *ex vivo* reactivation. In fact, *ex vivo* reactivation was not observed for any group which is in agreement with the literature: The frequency of splenocytes reactivating *ex vivo* strongly decreases between day 16 and 42 after infection with the practical consequence that there is hardly any reactivation detectable in the *ex vivo* reactivation assay at day 42 after infection^39^. These data indicate that the absence of the sncRNAs results in a higher viral genomic load in the spleen during late latency. To provide evidence that the sncRNAs are indeed expressed *in vivo* in latently infected mice, we exemplarily analysed the expression of mghv-mir-M1-1 in splenocytes of mice infected with parental virus. Consistent with previous data by Feldman *et al*.^45^, who analyzed miRNA expression from splenocytes 16 days after infection, we could readily demonstrate expression of mghv-mir-M1-1 both at days 16 and 43 after infection (Supplementary Figure S2).

### Initial analysis of the immune response in C57BL/6 wildtype mice

To get a first idea whether the deletion of the MHV-68 sncRNAs might affect the composition of lymphocyte subpopulations in the spleen or the humoral immune response after infection, spleens were harvested at day 17 after infection, and splenocytes were analyzed by FACS analysis (Supplementary Figure S3A). While no differences were observed for NK1.1-positive NK cells and for CD4- or CD8-positive T-cells, a significant reduction of CD19-positive B cells was oberved after infection with the sncRNA ko, when compared to infection with parental virus or sncRNA ko-revertant (P = 0.008 for parental virus versus sncRNA ko and for sncRNA ko versus sncRNA ko-revertant; Mann-Whitney Rank Sum Test). In addition, blood was collected at days 17 and 42 after infection, and MHV-68 specific antibodies in the sera were determined by ELISA (Supplementary Figure S3B). No differences in antibody titers between the groups were observed. We conclude from these data that the sncRNAs of MHV-68, consistent with the results concerning splenomegaly and *ex vivo* reactivation, contribute to the expansion of CD19-positive B cells at early latency.

### Lytic replication *in vivo* in IFN-γ-R^−/−^ mice

**Figure 7:**
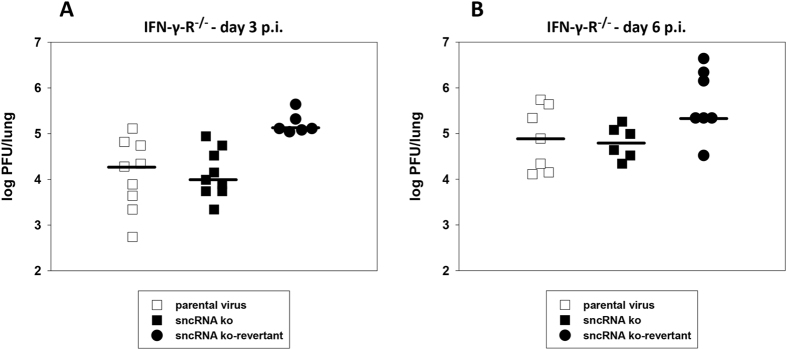
Lytic replication in the lungs of IFN-γ-R^−/−^ mice. Mice were inoculated i.n. with 5 × 10^4^ PFU of the indicated viruses. Lungs of mice were harvested at day 3 (**A**) and at day 6 (**B**) after infection. Virus titers were determined from organ homogenates by plaque assay. Each symbol represents an individual mouse, and the bars represent the median. The data are compiled from two to four independent experiments.

To further substantiate the results obtained in wildtype C57BL/6 mice in another model system, and since IFN-γ has been shown to be involved in limiting MHV-68 reactivation from latency^49^, we next analyzed the role of the MHV-68 sncRNAs during infection of IFN-γ-R^−/−^ mice. IFN-γ-R^−/−^ mice infected with wildtype MHV-68 are not different to wildtype mice when lytic virus titers in the lungs are regarded but display a marked loss of splenocytes (resulting in a shrunken spleen) and a greater load of latently infected cells in the spleen^50^,^51^. Thus, we first tested whether deletion of the MHV-68 sncRNAs has an effect on lytic replication in the lungs. For this purpose, IFN-γ-R^−/−^ mice were inoculated i.n. with 5 × 10^4^ PFU of parental virus, sncRNA ko and sncRNA ko-revertant, respectively. Lungs of mice were harvested at day 3 (Fig. 7A) and at day 6 (Fig. 7B) after infection, and virus titers were determined from organ homogenates by plaque assay. As in C57BL/6 mice, the sncRNA ko attained similar titers as parental virus and sncRNA ko-revertant (the latter displaying slightly higher titers), indicating that the sncRNAs are dispensable for lytic replication also in the lungs of IFN-γ-R^−/−^ mice.

### Early latency in IFN-γ-R^−/−^ mice

**Figure 8:**
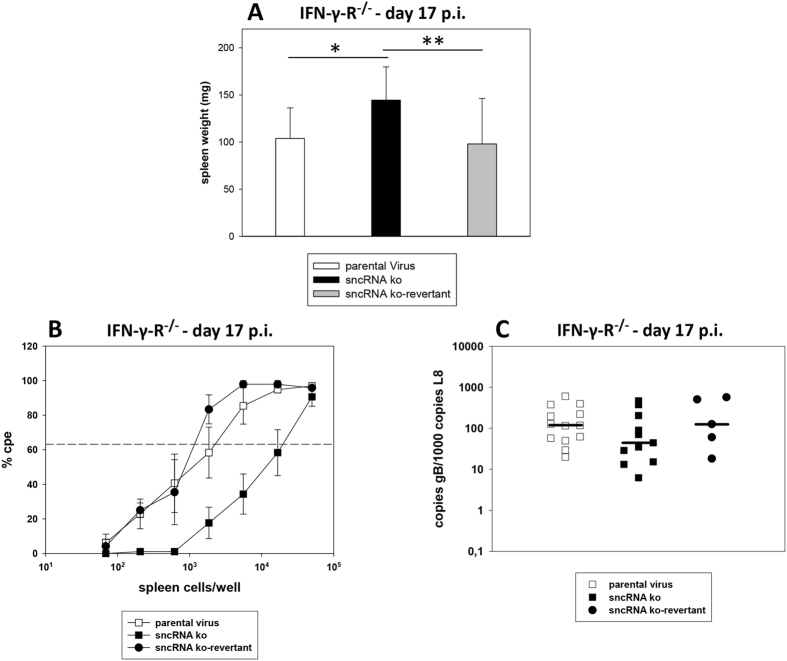
Early latency in the spleen of IFN-γ-R^−/−^ mice. Mice were inoculated i.n. with 5 × 10^4^ PFU of the indicated viruses. At day 17 after infection, spleens were harvested, and the spleen weights were taken. Single splenocyte suspensions were prepared and analyzed in the *ex vivo* reactivation assay or used for DNA isolation for real-time PCR analysis. (**A**) Spleen weights; (**B**) *ex vivo* reactivation of splenocytes; (**C**) viral genomic load in the spleen. Data shown in panel A are the means + standard deviations of 13 (parental virus), 11 (sncRNA ko) and 5 (sncRNA ko-revertant) mice per group, compiled from two to four experiments. The asterisks indicate a statistically significant difference: *P = 0.007; **P = 0.046 (Student’s t-test). Data shown in panel B are the means ± standard errors of the means pooled from two to four independent experiments. In each experiment, splenocytes from two to five mice per group were pooled. The dashed line in panel B indicates the point of 63.2% Poisson distribution, determined by nonlinear regression, which was used to calculate the frequency of cells reactivating lytic replication. In panel C, each symbol represents an individual mouse, and the bars represent the median. The data are compiled from two to four independent experiments.

Next, spleen weight, *ex vivo* reactivation of latently infected splenocytes and the viral genomic load in the spleen were determined 17 days after infection. To this end, IFN-γ-R^−/−^ mice were inoculated i.n. with 5 × 10^4^ PFU of parental virus, sncRNA ko and sncRNA ko-revertant, respectively. At day 17 after infection, spleens were harvested, and the spleen weights were taken. Single splenocyte suspensions were prepared and analyzed in the *ex vivo* reactivation assay or used for DNA isolation for real-time PCR analysis. After infection with the sncRNA ko, significantly higher spleen weights were observed, when compared to infection with parental virus or sncRNA ko-revertant (Fig. 8A). *Ex vivo* reactivation was approximately 10-fold reduced after infection with the sncRNA ko, with frequencies of reactivation being 1 in 2615, 1 in 20582 and 1 in 1274 for parental virus, sncRNA ko and sncRNA ko-revertant, respectively (P = 0.057 for parental virus versus sncRNA ko, and P = 0.133 for sncRNA ko versus sncRNA ko-revertant; Mann-Whitney Rank Sum Test) (Fig. 8B). In contrast, the viral genomic load in the spleen was not reduced after infection with the sncRNA ko (Fig. 8C). We conclude from these data that in IFN-γ-R^−/−^ mice, the sncRNAs of MHV-68 contribute to the loss of splenocytes and to *ex vivo* reactivation but not to the overall genomic load in the spleen at early latency.

### Late latency in IFN-γ-R^−/−^ mice

**Figure 9:**
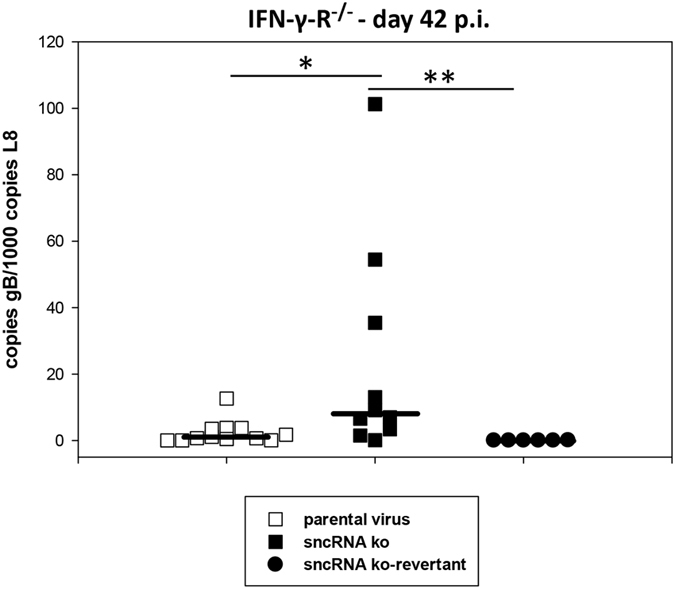
Late latency in the spleen of IFN-γ-R^−/−^ mice. Mice were inoculated i.n. with 5 × 10^4^ PFU of the indicated viruses. At day 42 after infection, spleens were harvested and used for DNA isolation for real-time PCR analysis to determine the viral genomic load. Each symbol represents an individual mouse, and the bars represent the median. The data are compiled from two to four independent experiments. The asterisks indicate a statistically significant difference: *P = 0.011; **P = 0.002 (Mann-Whitney Rank Sum test).

Finally, we analyzed the viral genomic load at day 42 post infection. Notably, as in C57BL/6 mice, IFN-γ-R^−/−^ mice infected with the sncRNA ko displayed a significantly higher viral genomic load when compared to infection with parental virus or sncRNA ko-revertant (P = 0.011 for parental virus versus sncRNA ko, and P = 0.002 for sncRNA ko versus sncRNA ko-revertant; Mann-Whitney Rank Sum Test) (Fig. 9). Again, as in C57BL/6 mice, the increase in viral genomic load was, however, not accompanied by an increased *ex vivo* reactivation. These data indicate that the absence of the sncRNAs results in a higher viral genomic load in the spleen during late latency.

### Pathogenesis in Balb/c IFN-γ^−/−^ mice

**Figure 10:**
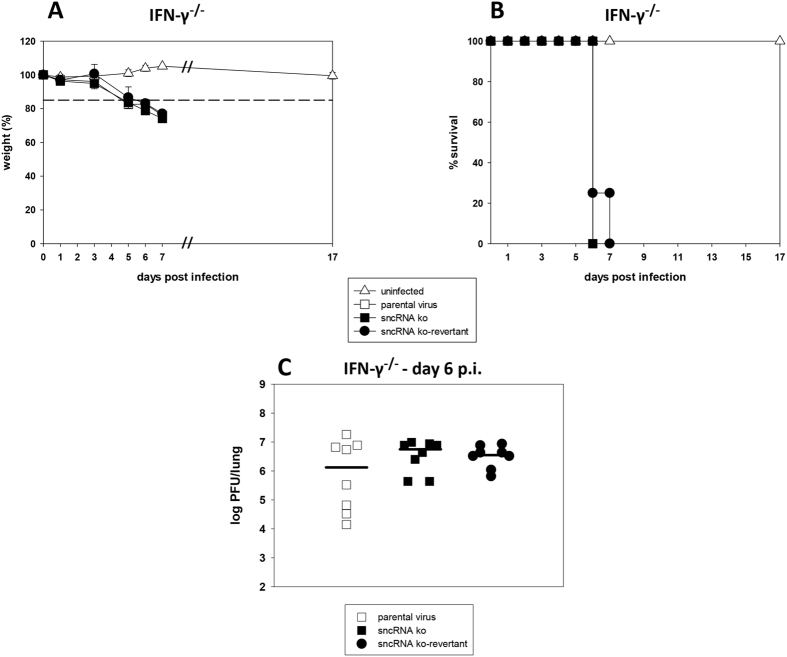
Pathogenesis in Balb/c IFN-γ^−/−^ mice. Mice were inoculated i.n. with 4 × 10^5^ PFU of the indicated viruses. Mice were monitored daily for signs of disease, and the body weight was determined at the indicated time points. Any mice that appeared moribund or lost more than 15% of the initial body weight (indicated by the dashed line) were sacrificed by cervical dislocation, and lungs were taken for further analyses. (**A**) weight loss; (**B**) survival; (**C**) virus titer in the lungs at day 6 after infection. Data shown in panels A (mean ± SD) and B are derived from 7 (uninfected), 11 (parental virus), 11 (sncRNA ko) and 11 (sncRNA ko-revertant) mice per group. The data are compiled from two independent experiments. In panel C, each symbol represents an individual mouse, and the bars represent the median.

Infection of Balb/c IFN-γ^−/−^ mice with wildtype MHV-68 results in acute lethal pneumonia^52^. Since other authors^45^,^46^ demonstrated that the MHV-68 sncRNAs are required for this lethal pneumonia, we also wanted to test our sncRNA ko in this system. For this purpose, Balb/c IFN-γ^−/−^ mice were inoculated i.n. with 4 × 10^5^ PFU of parental virus, sncRNA ko and sncRNA ko-revertant, respectively, or were left uninfected. Subsequently, mice were monitored daily for signs of disease and the body weight was determined. Any mice that appeared moribund or lost more than 15% of the initial body weight were sacrificed by cervical dislocation, and lungs were taken for further analyses. As shown in Fig. 10, no differences regarding weight loss (Fig. 10A), survival (Fig. 10B) and viral lung titers at day 6 after infection (Fig. 10C) were observed between the mice infected with the different viruses.

## Discussion

In MHV-68, the miRNAs are located downstream of the vtRNAs. For this reason, these very tightly linked elements are also called TMERs (tRNA-miRNA-encoding RNAs)^45^,^46^. A practical consequence is that it might be inherently difficult to separate potential functions of the vtRNAs and miRNAs from each other. A first attempt in this direction was recently undertaken by Diebel *et al*.^46^, suggesting an independent functional role of the vtRNAs in acute infection and pathogenesis. Yet, in general, uncovering the functions of the vtRNA-miRNA-encoding sequences is still in its beginning.

Consequently, at this stage, we decided to investigate the effect of a combined deletion of both vtRNAs and miRNAs using a mutant lacking both types of sncRNAs. The data obtained with our mutant are mostly consistent with the previous findings of Feldman *et al*.^45^ and Diebel *et al*.^46^. Specifically, we also observed that the sncRNAs of MHV-68 are dispensable for lytic replication both vitro and *in vivo*. In contrast, at early latency in C57BL/6 wildtype mice, a mutant lacking the sncRNAs was slightly attenuated as reflected by reduced spleen weight, reduced B cell expansion and reduced *ex vivo* reactivation. Interestingly, this was not accompanied by a reduced overall viral genomic load. We also investigated our mutant in IFN-γ-R^−/−^ mice, thereby extending the previous studies to an additional *in vivo* model. In IFN-γ-R^−/−^ mice, we observed similar results as in C57BL/6 wildtype mice, thus validating the findings.

Surprisingly, in Balb/c IFN-γ^−/−^ mice, we obtained completely different results when compared to the previous studies. While Feldman *et al*.^45^ and Diebel *et al*.^46^ observed a striking attenuation of lethal pneumonia in Balb/c IFN-γ^−/−^ mice after infection with their respective mutants, we did not observe a reduced virulence of our mutant in this model of viral pneumonia^52^. However, we think that this difference can most likely be explained by the fact that we, according to our national animal welfare rules, sacrificed the animals when they lost more than 15% of the initial body weight. We assume that in the studies of Feldman *et al*.^45^ and Diebel *et al*.^46^, the mice got the opportunity to recover.

Most importantly, while we, like Feldman *et al*.^45^ and Diebel *et al*.^46^, also investigated acute lytic replication and early latency, we additionally analyzed late latency (around six weeks after infection). This appeared very interesting to us as it has been proposed that the use of ncRNAs might be of particular advantage for the virus during tight latency. At this stage, hardly any viral proteins are expressed to avoid recognition by the immune system, and thus, ncRNAs might be exploited since they are less likely to be recognized by the immune system^2^. In particular, it has been proposed that ncRNAs directly or indirectly contribute to the maintenance of latency, either by targeting pivotal viral lytic genes or by targeting cellular regulatory pathways used to control latency^16^,^19^,^20^,^53^,^54^. For gammaherpesviruses, this has been shown for KSHV where viral miRNAs help to maintain latency by mediating the repression of RTA (replication and transcription activator) or by targeting the NF-kB pathway, and it has also been shown for EBV where viral miRNAs help to maintain latency by targeting BALF5 (the EBV DNA polymerase) or by targeting Dicer (see references 12, 18, 19, 53, 54, 55 for recent reviews). The studies with KSHV and EBV have all been performed *in vitro*. Therefore, until now, no information was available whether gammaherpesviral sncRNAs may also contribute to the maintenance of latency *in vivo*. We investigated this issue by analyzing the phase of late latency (around six weeks after infection) in the MHV-68 model.

In the absence of the sncRNAs, we observed a higher viral genomic load during late latency in the spleens of both C57BL/6 wildtype and IFN-γ-R^−/−^ mice. We speculate that the increased viral genomic load during late latency is due to a disturbed regulation of the balance between latent and lytic infection. The increase in viral genomic load was not accompanied by an increased *ex vivo* reactivation. However, it is known that at six weeks post infection, only very few latently infected splenocytes reactivate in the *ex vivo* reactivation assay^39^. At the moment, we do neither understand the molecular mechanisms nor do we know whether the observed effects are related to the MHV-68 miRNAs, to the vtRNAs or to a combination of both. Clearly, much more work with more sophisticated mutants will be necessary to separate the potential functions of the vtRNAs and miRNAs from each other. In this regard, Feldman *et al*.^56^ very recently showed for the first time a miRNA-independent function of a MHV-68 TMER: TMER4 was shown to be important for hematogenous dissemination of MHV-68 and for the establishment of latency at peripheral sites. In addition, to understand the regulation exerted by the sncRNAs, in particular by the miRNAs, we need to know potential targets of the MHV-68 miRNAs. Until now, only a bioinformatical prediction of putative targets has been performed but no targets have been validated so far^57^.

Taken together, we demonstrated that during late latency, the absence of the MHV-68 sncRNAs results in a higher viral genomic load in the spleen. Hence, we provide for the first time evidence that gammaherpesvirus sncRNAs contribute to the maintenance of latency *in vivo*.

## Methods

### Cell lines

BHK-21 cells (American Type Culture Collection [ATCC]: CCL-10) were grown in Glasgow-MEM (PAN Biotech, Aidenbach, Germany) supplemented with 5% fetal calf serum (FCS; PAN Biotech, Aidenbach, Germany), 5% Tryptose Phosphate Broth, 2 mM L-Glutamine, 100 U/ml Penicillin and 100 μg/ml Streptomycin. REF-Cre cells^47^ were maintained in DMEM High Glucose (Gibco, Darmstadt, Germany) with 10% FCS, 2 mM L-Glutamine, 100 U/ml Penicillin, 100 μg/ml Streptomycin and 500 μg/ml G418 (PAA, Pasching, Austria). NIH3T3 cells (ATCC: CRL-1658) were grown in DMEM High Glucose (Gibco, Darmstadt, Germany) in the presence of 10% FCS, 2 mM L-Glutamine, 100 U/ml Penicillin and 100 μg/ml Streptomycin.

### Plasmid construction and analysis of BAC plasmids

Using MHV-68 cloned as bacterial artificial chromosome (BAC) (designated as parental virus)^47^, we constructed a mutant lacking all known sncRNAs (named as sncRNA ko) and a revertant of this mutant (called as sncRNA ko - revertant) as follows:

1. The mutant virus sncRNA was created by three consecutive rounds of markerless BAC mutagenesis as described previously^58^. First, nucleotides 196 to 1788 were deleted (thereby deleting tRNAs 1–5 including miRNAs 1–7 and 10–12) using the forward primer 5′-TAG AGC AAC AGG TCA CCG ATC CTG GTG GTT CTC GGT TCA AGT CCG AGC TCA GGA TGA CGA CGA TAA GTA GGG-3′ and the reverse primer 5′-TGT TCT TGT TTT GGT GGG AGG CTA GAT GGT CGT GGG CGG CCT GTG GAG CAG AGC TCG GAC TTG AAC CGA GAA CCA CCA GGA TCG GTG ACC TGT TGC TCT ACA ACC AAT TAA CCA ATT CTG ATT AG-3′. Second, nucleotides 3749 to 3876 were deleted (thereby deleting miRNAs 8 and 13 and possibly also affecting tRNA 6) using the forward primer 5′-GCA GCG GCC ACC AAG CCT GCA GGT TCT CGG TTC AAG TCC GGG CGC TGG CAA GGA TGA CGA CGA TAA GTA GGG-3′ and the reverse primer 5′-ACA CTC TTT TAC AAC CCT AGG AGA GCC AGA TAA AAA TTT TGT GTT AAG CAT GCC AGC GCC CGG ACT TGA ACC GAG AAC CTG CAG GCT TGG TGG CCG CTG CCA ACC AAT TAA CCA ATT CTG ATT AG-3′. Finally, nucleotides 5102 to 5584 were deleted (thereby deleting miRNA14 and tRNA 8 including miRNAs 9 and 15, and possibly also affecting tRNA7) using the forward primer 5′-CAA ACT GTG TGG TGG GAC TCT GCA GAC TTG GCA GTT CTG CAG CAG TCA GCA GGA TGA CGA CGA TAA GTA GGG-3′ and the reverse primer 5′-GTG TCC TAA ATT CTG AGG CCA ATG GAA AGG TGA GCA TGT CTT GGG TGG CAG CTG ACT GCT GCA GAA CTG CCA AGT CTG CAG AGT CCC ACC ACA CAG TTT GCA ACC AAT TAA CCA ATT CTG ATT AG-3′.
2. To exclude the possibility that the observed results were due to rearrangements outside the mutated regions, a revertant of the sncRNAs ko mutant (sncRNA ko - revertant) was generated by the two-step mutagenesis procedure^47^,^59^. For that purpose, a 1783 bp HindIII - StuI fragment of MHV-68 (nucleotide positions 106 to 1889) was cloned blunt end into the BglII site (nucleotide position 3846 of the MHV-68 genome) of the plasmid pST76K-SR M1/M2^60^. As a result, the 1783 bp fragment is flanked on both sides by homologous sequences (position 2406–3846 as 5′ flank and position 3847–6261 as 3′ flank) as needed for homologous recombination during the two-step mutagenesis procedure.

All BAC plasmids were characterized by restriction enzyme and Southern blot analysis and by sequencing across the mutated regions.

### Reconstitution of recombinant MHV-68

To reconstitute recombinant MHV-68, BHK-21 cells were transfected with 2 μg of the appropriate BAC MHV-68 DNA using X-treme GENE HP DNA Transfection Reagent (Roche, Mannheim, Germany). When cells showed a total cytopathic effect (CPE), an aliquot of the supernatant was used to infect REF-Cre cells carrying the Cre recombinase to remove the BAC-cassette including the GFP sequence. BAC-cassette free viruses were identified by a limiting dilution assay on BHK-21 cells performed in a 96 well plate. All recombinant viruses were grown and titrated on BHK-21 cells as previously described^47^.

### Determination of viral gene expression by RT-PCR

NIH3T3 cells were cultured in 6-well plates and infected with the indicated viruses at an MOI of 1. For each virus, three biological replicates were done. Total RNA was isolated 48 hr p.i. using the RNeasy MiniKit (Qiagen, Hilden, Germany). Genomic DNA was removed with the TURBO DNA-free Kit (Ambion/Life Technologies, Darmstadt, Germany), and RNA was reverse-transcribed using the High Capacity cDNA Reverse Transcription Kit (Applied Biosystems, Foster City, CA) or subjected to mock reverse transcription in the absence of the enzyme (-RT control). The expression of the MHV-68 genes M1, M2 and M3 and of the cellular ribosomal Protein L8 gene was analyzed by real time quantitative PCR using Taqman SYBR green PCR master mix (Applied Biosystems, Foster City, CA) and the ABI 7300 Real Time PCR System (Applied Biosystems, Foster City, CA). The following PCR primer sets were used: L8: forward 5′-CAG TGA ATA TCG GCA ATG TTT TG-3′; reverse 5′-TTC ACT CGA GTC TTC TTG GTC TC-3′. M1: forward 5′-ATC TCA CCT TTG CTG GAT TCT TAT TTG C-3′; reverse 5′-GTT CTG ATG GCT TGA AAC GAT GGC-3′. M2: forward 5′-TCC TCG CCC CAC TCC ACA AAA C-3′; reverse 5′-AAC ACC CCA TGA ACC CTG AGA TAC G-3′. M3: forward 5′-GCT TGC TGG ATG GTC TCA CAG G-3′; reverse 5′-TAA GAT GAA CAC TTG CCC ATG CTA CTA C-3′.

### Determination of miRNA expression by the recombinant MHV-68

NIH3T3 cells were cultured in 6-well plates and infected with the indicated viruses at an MOI of 1, or were left uninfected. For each condition, three biological replicates were done. Total RNA was isolated 48 hr p.i. using the RNAeasy Mini-Kit (Qiagen, Hilden, Germany) according to the instructions of the manufacturer. RNA samples were then submitted to LC Sciences (Houston, Texas, USA). The expression analysis of the MHV-68 miRNAs was performed by LC Sciences using custom miRNA microarrays.

For samples derived from latently infected mice, quantification of miRNAs was performed as previously described by stemloop quantitative RT-PCR (qRT-PCR)^41^.

### Analysis of *in vitro* growth properties of the recombinant MHV-68

To test *in vitro* growth of the virus mutants, NIH3T3 cells were infected with an MOI of 0.1 for 1 hour. After removing the inoculum, cells were incubated with fresh medium at 37 °C and 5% CO_2_ until the supernatants together with the cells were harvested at different time points after infection. Virus titers were determined by plaque assay.

### *In vivo* experiments

C57BL/6 mice were purchased from Charles River Laboratories (Sulzfeld, Germany). IFN-γ-R^−/−^ mice (on C57BL/6 background) and IFN-γ^−/−^ mice (on Balb/c background) were originally obtained from the Jackson Laboratory (Bar Harbor, Maine, USA) and subsequently bred and propagated under SPF conditions at the Helmholtz Zentrum München. Mice were housed in individually ventilated cages (IVC) during the MHV-68 infection period. To characterize the recombinant MHV-68 *in vivo*, mice were infected i.n. with 5 × 10^4^ PFU. Prior to i.n. infection, mice were anesthetized with ketamine-xylazine or with medetomidine-midazolam-fentanyl. To determine virus titers, organs were harvested at the indicated time points after infection and homogenized by using the FASTPREP^®^-24 instrument (MP Biomedicals, Heidelberg, Germany). After two times freezing and thawing the homogenates, plaque assays were performed with 10-fold dilutions of the supernatants on BHK-21 cells. For the measurement of spleen weight, frequency of virus reactivation and genomic load as well as for FACS analysis, spleens were harvested at the indicated time points after infection. For the determination of MHV-68 specific antibodies, blood was collected at the indicated time points after infection. Mice were monitored daily for signs of disease, and any mice that appeared moribund were sacrificed by exposure to CO_2_ or by cervical dislocation.

### Ethics Statement

All animal experiments were in compliance with the German Animal Welfare Act (German Federal Law §8 Abs. 1 TierSchG), and the protocols were approved by the local Animal Care and Use Committee (District Government of Upper Bavaria; permit number 124-08 and 154-13).

### Limiting dilution reactivation assay

To determine the frequency of cells carrying virus reactivating from latency, threefold dilutions of splenocytes were plated onto NIH 3T3 cells as described previously^61^. Frequencies of reactivating cells were calculated on the basis of the Poisson distribution by determining the cell number at which 63.2% of the wells scored positive for CPE.

### Measurement of latent viral load by real time PCR

Viral load in the spleens of infected mice was determined by quantitative real-time PCR using the ABI 7300 Real Time PCR System (Applied Biosystems, Foster City, CA) as described previously^62^.

### Determination of MHV-68 specific antibodies

To quantify MHV-68 specific antibodies in sera of mice, an ELISA was performed as described by Ruiss *et al*.^63^. Briefly, Nunc Maxisorp plates (Nunc, Wiesbaden, Germany) were coated with 3 μg/ml of MHV-68 lysate (prepared by disrupting MHV-68 via dilution in PBS/0.05% Triton X-100) for 90 min at 37 °C and stored overnight (4 °C). After washing with PBS/Tween, plates were blocked with PBS/Tween containing 5% FCS for 1 hour and washed again. Diluted sera (1:50) including positive and negative control sera were incubated for 1 h. For each sample, triplicates were performed. After washing, bound antibody was detected with HRP-conjugated rat anti-mouse antibody (Promega, Mannheim, Germany) and TMB (Becton Dickinson, Heidelberg, Germany) as substrate. Reactions were stopped with 1 M phosphoric acid and absorbance was read at 450 nm (Tecan Sunrise, Switzerland).

### FACS analysis

The following fluorescent dye-conjugated anti-mouse monoclonal antibodies (mAbs) were used to evaluate and quantify amounts and phenotypes of splenocytes after infection by FACS: CD3-FITC (clone: 17A2), CD4-PE (RM4-5), CD8a-PE (53-6.7), CD19-PE (PeCa1) and NK1.1-PE (PK136). mAbs were purchased from either BD/Pharmingen (Heidelberg, Germany) or ImmunoTools (Friesoythe, Germany). Cells were incubated with mAbs (PBS + 2% FCS) according to manufacturer’s instructions, including appropriate isotype controls. Data were evaluated on a FACS-Calibur-Flow-Cytometer using Cell-Quest-data acquisition and analysis software (BD).

### Statistical methods

Datasets were tested for Gaussian distribution before statistical analysis by Shapiro-Wilk Normality Test using the SigmaPlot 12 software (Systat Software GmbH, Erkrath, Germany). When passing the normality test, data were analyzed by Student’s t-test. All other datasets were analyzed by Mann-Whitney Rank Sum Test. Results with a p-value < 0.05 were considered significant.

## Additional Information

**How to cite this article**: Steer, B. *et al*. The small noncoding RNAs (sncRNAs) of murine gammaherpesvirus 68 (MHV-68) are involved in regulating the latent-to-lytic switch *in vivo.* *Sci. Rep.* **6**, 32128; doi: 10.1038/srep32128 (2016).

## Supplementary Material

Supplementary Information
