# Supplementary material for: The small noncoding RNAs (sncRNAs) of murine gammaherpesvirus 68 (MHV-68) are involved in regulating the latent-to-lytic switch in vivo
Source: Sci Rep. 2016 Aug 26;6:32128. doi: 10.1038/srep32128 (PMC4999806; doi:10.1038/srep32128)
Supplement: Supplementary Information [file srep32128-s1.pdf]

Supporting Information

The small noncoding RNAs (sncRNAs) of murine gammaherpesvirus 68 (MHV-68) are involved in regulating the latent-to-lytic switch *in vivo*

Beatrix Steer [1], Martin Strehle [1], Christine Sattler [1, 2], Dagmar Bund [1], Britta Flach [1], Tobias Stoeger [2], Jürgen G. Haas [3], and Heiko Adler [1]

[1] Comprehensive Pneumology Center, Research Unit Lung Repair and Regeneration, Helmholtz Zentrum München - German Research Center for Environmental Health (GmbH), Marchioninistrasse 25, D-81377 Munich, Germany, and University Hospital Grosshadern, Ludwig-Maximilians-University, Munich, Germany; Member of the German Center of Lung Research (DZL)

[2] Institute of Lung Biology and Disease, Comprehensive Pneumology Center, Helmholtz Zentrum München - German Research Center for Environmental Health (GmbH), Ingolstädter Landstr. 1, D-85764 Neuherberg, Germany

[3] Division of Infection and Pathway Medicine, University of Edinburgh, 49 Little France Crescent, Edinburgh EH16 4SB, UK

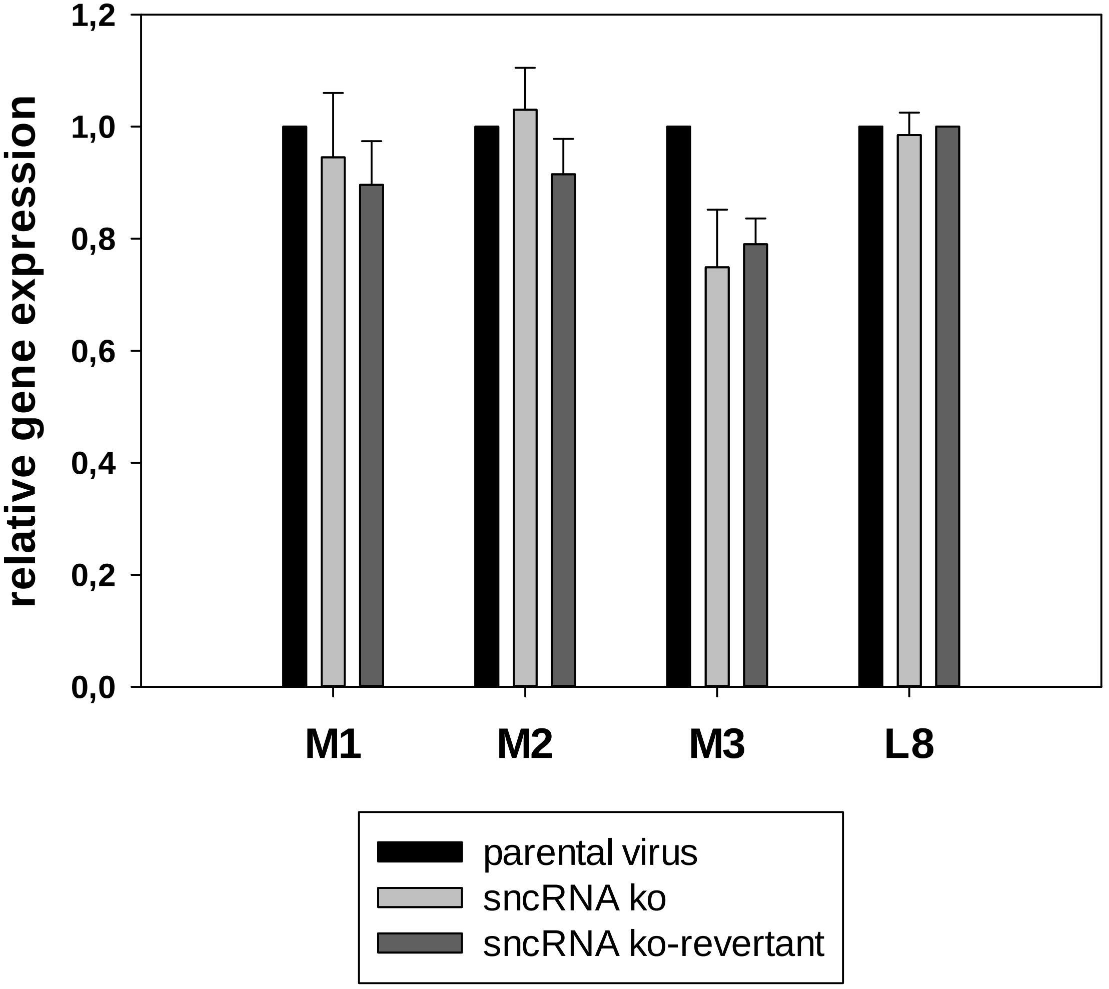

1,2
1,0
0,8
0,6
0,4
0,2
0,0
relative gene expression
M1
M2
M3
L8
parental virus
sncRNA ko
sncRNA ko-revertant

Supplementary Figure S1: Gene expression of neighbouring genes. Transcription of M1, M2 and M3 was determined 48 hours after infection by quantitative RT-PCR in NIH3T3 cells infected with the indicated viruses. Shown are means + SD of three biological replicates. L8 transcription was analyzed in parallel as a control.

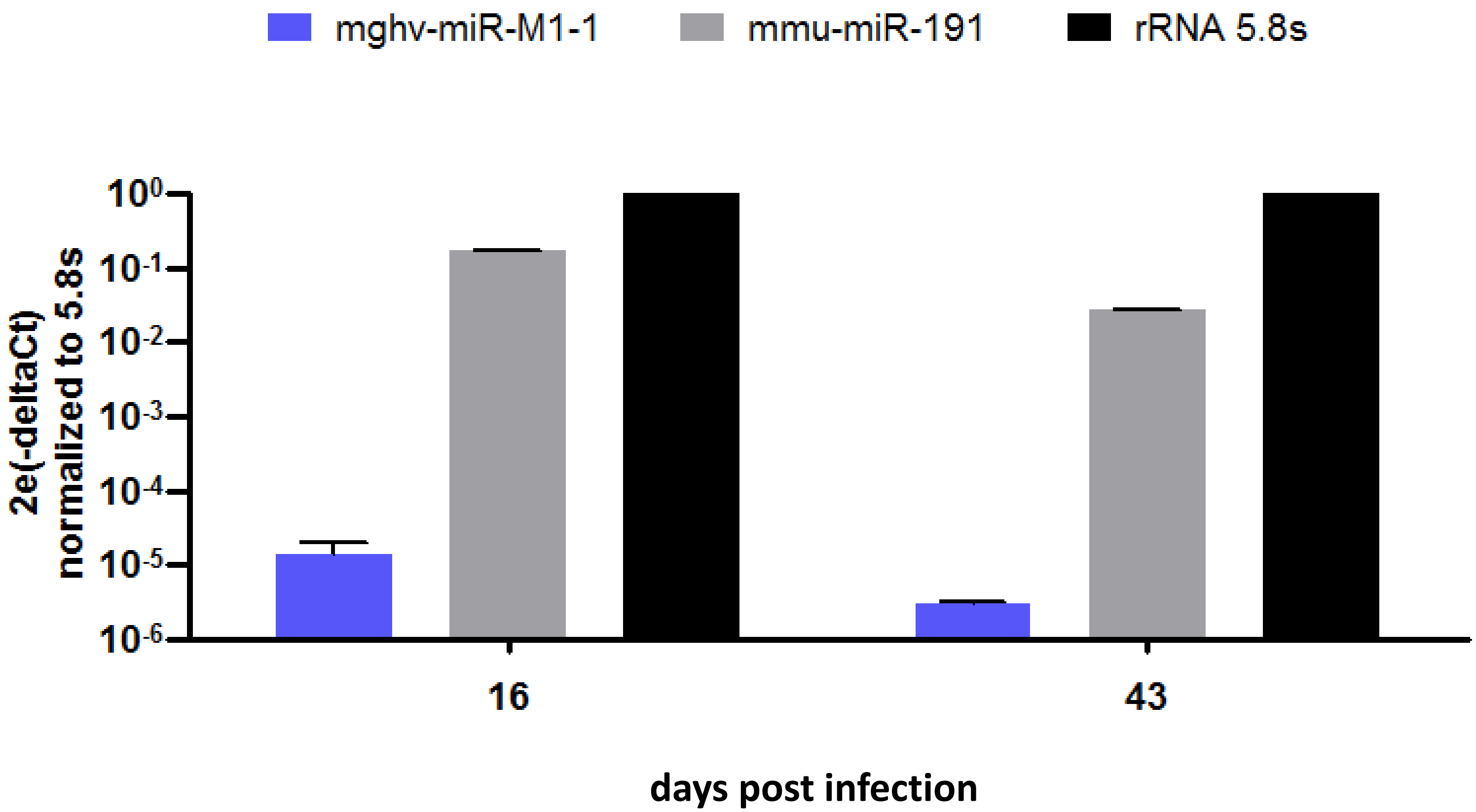

mghv-miR-M1-1
mmu-miR-191
rRNA 5.8s
2e(-deltaCt)
normalized to 5.8s
10^0
10^-1
10^-2
10^-3
10^-4
10^-5
10^-6
16
43
days post infection

Supplementary Figure S2: Expression of mghv-mir-M1-1 in vivo. RNA was extracted from splenocytes of mice infected with parental virus at the indicated time points after infection and subjected to stem-loop RT-qPCR as described in Materials and Methods. As a positive control, the expression of the cellular miRNA mmu-miR-191 is also shown. Data shown are means + SD (n=2).

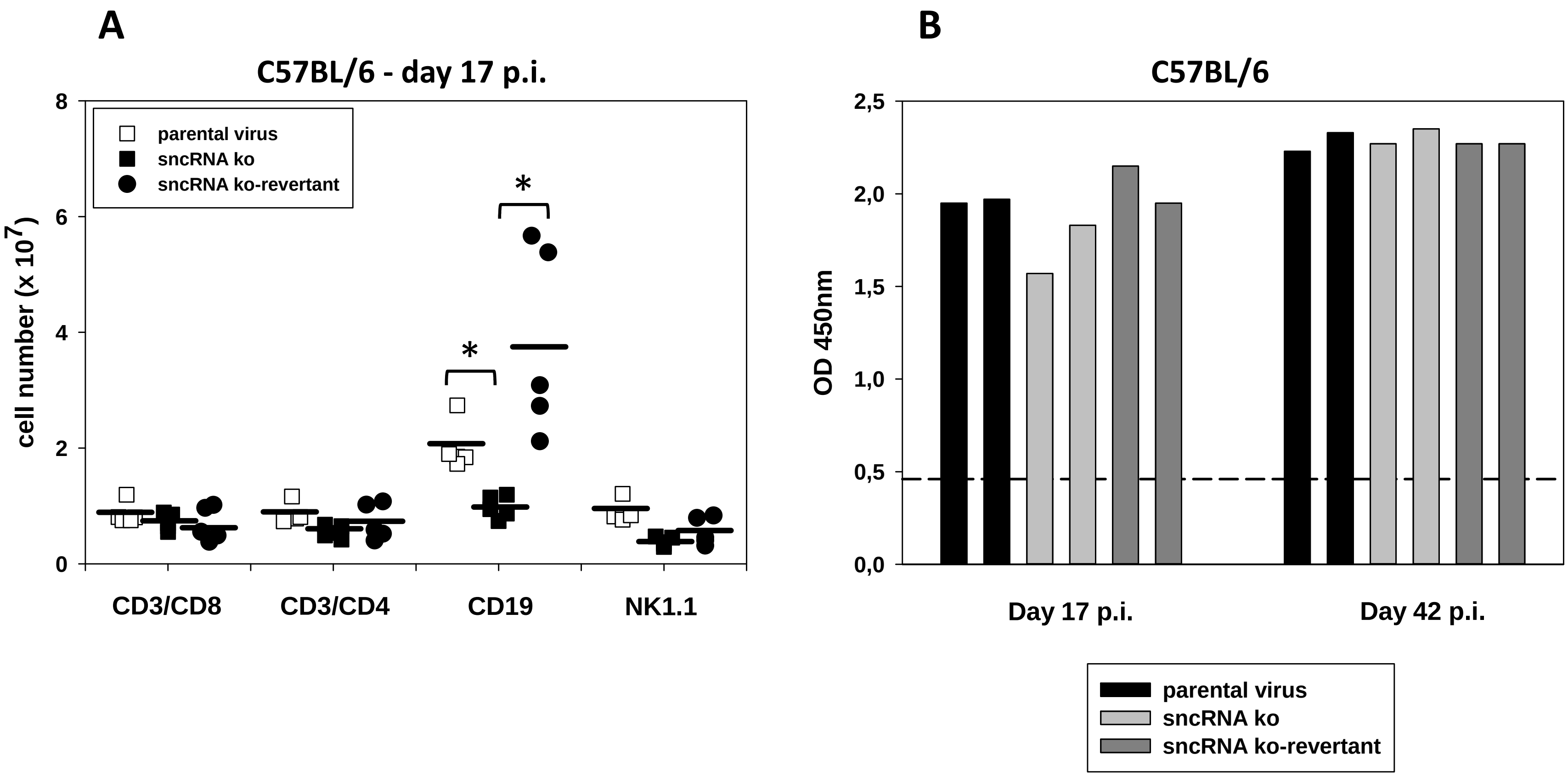
A
C57BL/6 - day 17 p.i.
parental virus
sncRNA ko
sncRNA ko-revertant
cell number (x 10^7)
0
2
4
6
8
*
*
CD3/CD8
CD3/CD4
CD19
NK1.1
B
C57BL/6
OD 450nm
0,0
0,5
1,0
1,5
2,0
2,5
Day 17 p.i.
Day 42 p.i.
parental virus
sncRNA ko
sncRNA ko-revertant

Supplementary Figure S3: Analysis of the immune response after infection. Mice were inoculated i.n. with $5x10^4$ PFU of the indicated viruses. A) At day 17 after infection, spleens were harvested, and single splenocyte suspensions were prepared and analyzed by FACS analysis. Each symbol represents an individual mouse, and the bars represent the mean. The asterisks indicate a statistically significant difference: * P = 0.008 (Mann-Whitney Rank Sum test). B) At days 17 and 42 after infection, blood was collected, and MHV-68 specific antibodies in the sera were determined by ELISA. The dashed line indicates the limit of detection.
